# Molecular and cellular mechanisms of muscle–adipose tissue crosstalk driving sarcopenic obesity: an integrative review of human, animal, and in vitro models

**DOI:** 10.1186/s12986-026-01123-2

**Published:** 2026-05-13

**Authors:** Mahmoud A. Seliem, Mamdouh A. Ragab

**Affiliations:** Department of Biochemistry, Faculty of Pharmacy, Ahram Canadian University, 6th of October City, Giza, Egypt

**Keywords:** Sarcopenic obesity, Muscle-adipose crosstalk, Anabolic resistance, GLP-1 receptor agonists

## Abstract

Sarcopenic Obesity is a complex geriatric syndrome that is not simply the co-occurrence of sarcopenia and obesity but is a synergistic pathophysiological relationship in which the sum of the risk factors is greater than the sum of the individual conditions. This integrative review proposes that sarcopenic obesity is fundamentally an illness of impaired bidirectional communication between skeletal muscle and adipose tissue that function as dynamic endocrine organs that interact through a vast repertoire of bioactive molecules. We critically analyze the molecular signaling pathways responsible for this crosstalk and the simultaneous hyperactivation of the Ubiquitin-Proteasome System and NF-κB pathways. Furthermore, highlighting the paradigm-shifting role of the Exosomal miRNA Regulatory Network, we explored how adipose-derived extracellular vesicles deliver atromiRs (e.g., miR-27a, Let-7d-3p) to myocytes and cause insulin resistance and regenerative failure, and the loss of myocyte-derived miR-146a-5p eliminates an important brake on adipogenesis. Also, we explored cellular mechanisms such as macrophage phenotypic switching (metaflammation), mitochondrial lipotoxicity, and the newly emerging gut-microbiota-muscle axis. Finally, we explored advanced experimental models, such as organ-on-a-chip systems, and explored the clinical paradox of GLP-1 receptor agonist-induced muscle loss. We conclude that future therapeutic frontiers are in the area of combination therapies and miRNA mimics to reinstate this broken molecular dialogue.

## Introduction

### The converging epidemics of sarcopenia and obesity

The world-wide demographic trend of ageing population and the increasing prevalence of obesity have led to the development of a complex geriatric syndrome called Sarcopenic Obesity (SO). This is a clinical entity characterized not only by the simultaneous occurrence of sarcopenia, or age-related loss of skeletal muscle mass, strength, and function, and obesity but also a synergistic pathophysiological relationship in which the combination of risk factors is greater than either condition alone. The condition is linked to a sudden decrease in physical performance, fall and fracture risks, metabolic imbalance and an increase in mortality [1].

In May 2025, the Asia-Oceania Association to study obesity published the Asia-Oceania Consensus: Definitions and Diagnostic Criteria to SO, which was a significant step towards standardizing the diagnosis of various populations [1]. Nevertheless, the etiology is still under active research. Unlike simple obesity, where skeletal muscle often undergoes a compensatory hypertrophy to bear increased mechanical loads [2], or pure sarcopenia, which is primarily driven by intrinsic aging factors like motor unit loss [3], SO represents a unique pathological entity. In SO, the lipotoxic and pro-inflammatory crosstalk from hypertrophic adipose tissue completely overwhelms the musculoskeletal system’s compensatory adaptations, forcefully shifting the muscle into a catabolic state [4].

This review proposes a unifying mechanistic framework for SO based on a distinct signaling hierarchy. The initiating driver is adipose tissue expansion, which creates a lipotoxic and pro-inflammatory environment. This dysfunction is systematically communicated as a systemic mediator to skeletal muscle via the circulation of pro-inflammatory adipokines, macrophage infiltration, and extracellular vesicles (EVs). The downstream consequence is intramyocellular lipid accumulation and inflammatory signaling that converge to uncouple the insulin pathway (anabolic resistance) and hyperactivate the Ubiquitin-Proteasome System, culminating in targeted muscle atrophy and a feed-forward loop of metabolic decline.

## Molecular signaling pathways: the engines of crosstalk

SO progression is supported by the impairment of the regulation of essential intracellular signaling pathways controlling protein turnover, mitochondrial biogenesis and inflammation.

### The insulin/IGF-1/PI3K/Akt/mTOR axis

The Insulin-like Growth Factor 1 (IGF-1) and insulin pathways play a critical role in the preservation of skeletal muscle mass. When bound by the ligand, the insulin receptor triggers Insulin Receptor Substrate-1 (IRS-1), which triggers Phosphoinositide 3-kinase (PI3K) and then Akt (Protein Kinase B). Phosphorylated Akt triggers the mechanistic Target of Rapamycin (mTOR) complex 1 (mTORC1) that mediates protein synthesis through p70S6 Kinase (p70S6K), and Eukaryotic Translation Initiation Factor 4E-Binding Protein 1 (4E-BP1) [5, 6]. At the same time, Akt phosphorylates Forkhead box O (FoxO) transcription factors which are sequestered in the cytoplasm and inhibit atrogenes transcription [7]. But in the sarcopenic obese condition, this pathway is suppressed on a long-term basis. Kinases like IKK, JNK are activated by pro-inflammatory cytokines (e.g. TNF-α), free fatty acids and phosphorylate IRS-1 at serine residues (e.g. Ser307); inhibition of physiological tyrosine phosphorylation of the receptor uncouples the receptor to PI3K [8]. This induced anabolic resistance inhibits muscle protein synthesis despite nutrient presence whereas the resultant de-repression of FoxO induces proteolysis [7].

### The ubiquitin-proteasome system and NF-κB

The hyperactivation of the Ubiquitin-Proteasome System is the major pathway of muscle wasting in SO. Nuclear Factor Kappa B (NF-κB) pathway is the key mediator of this atrophy caused by inflammation. IκB kinase (IKK) phosphorylates the inhibitor IKKα in response to adipose-derived TNF-α, which triggers its degradation to release NF-κB dimers (p50/p65) and translocate to the nucleus [8]. NF-κB, after activation, increases the expression of muscle-specific E3 ubiquitin ligases, namely, Muscle RING-finger protein-1 (MuRF1) and Muscle Atrophy F-box (MAFbx/Atrogin-1). These ligases mark myofibrillar proteins to be degraded by the 26 S proteasome [7]. To make this regulation more complex, the buildup of Let-7d-3p miRNA in aged perimuscular adipose tissue EVs has been found to reduce NF-κB signaling in adipocytes, but its transfer to muscle stem cells inhibits regeneration, which is an example of a nuance: NF-κB modulation has tissue-specific, deleterious effects [9].

### The myostatin/smad pathway

**Fig. 1:**
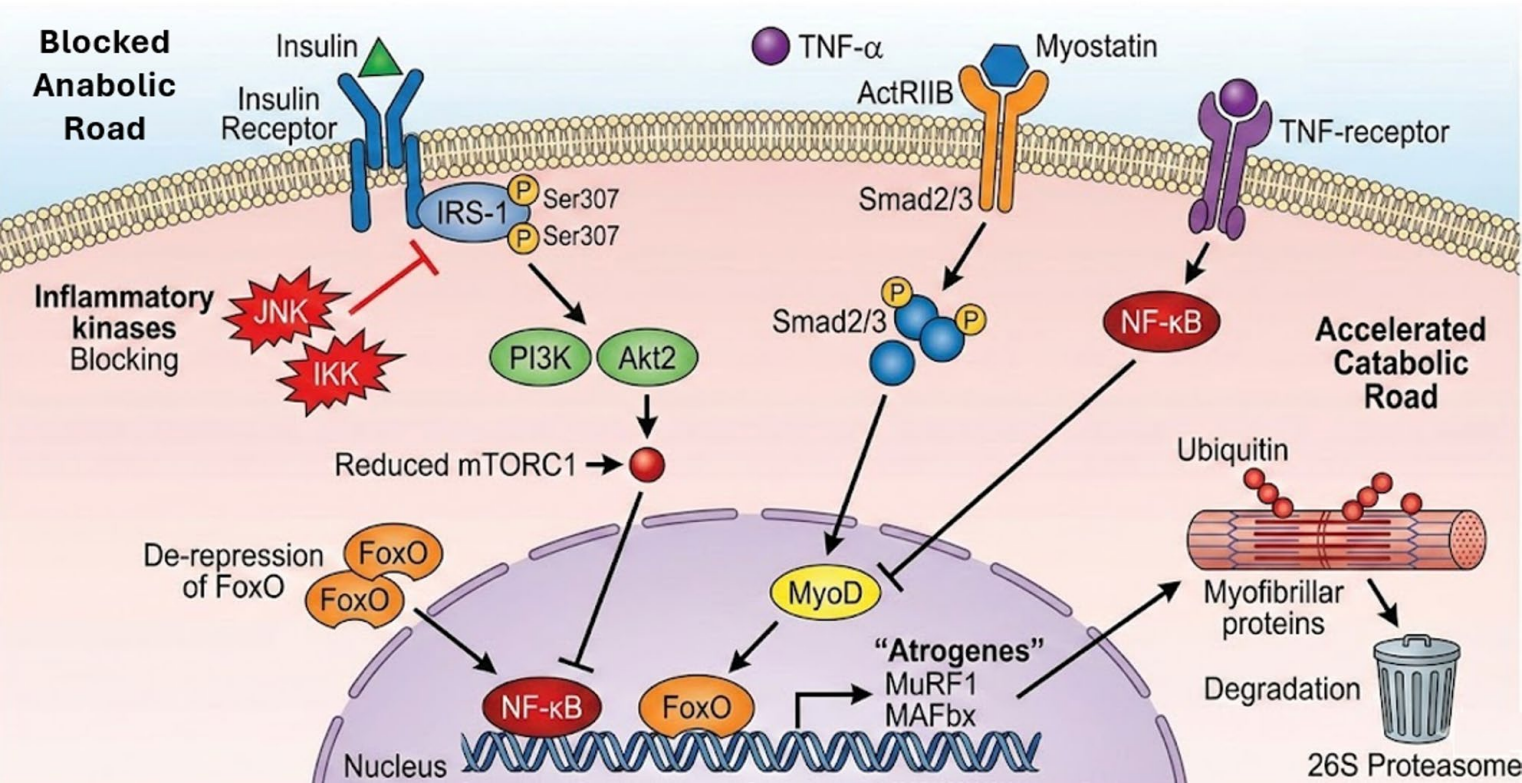
The “Anabolic Resistance” Intracellular Map

Myostatin is a TGF-b superfamily protein and is a strong negative regulator of muscle mass, which is a key factor in the pathology of SO. Mechanistically, myostatin interacts with the Activin Receptor type IIB (ActRIIB) and recruits ALK4/5 mechanoreceptors; the receptor complex then phosphorylates Smad2 and Smad3, which then heteromerise with Smad4 and translocate to the nucleus. Afterwards, Smad2/3 suppresses the expression of key myogenic regulatory factors, including MyoD and Myogenin, and collaborates with FoxO to activate atrogenes. Within the context of SO, the levels of serum myostatin are often increased and positively correlated with insulin resistance and negatively with skeletal muscle mass [10, 11]. It is important to note that myostatin acts as a dual-effector of the SO phenotype: in addition to muscle-wasting functions, it also suppresses the activity of AMPK in adipose tissue, thus inhibiting lipolysis and actively stimulating adiposity [12] Fig. 1.

### The JAK/STAT pathway

The Janus Kinase/Signal Transducer and Activator of Transcription (JAK/STAT) signaling cascade, which is mainly triggered by cytokines like interleukin-6 (IL-6) and Interferon- γ, is involved in a subtle role in SO. Although a transient IL-6 signaling may be metabolically advantageous, its sustained expression in obesity triggers JAK1/2 and STAT3. Continuous phosphorylation of STAT3 in skeletal muscle induces atrophy through the increases of Caspase-3 and the Ubiquitin-Proteasome System. This pathway is suppressed by the Suppressors of Cytokine Signaling (SOCS1 and SOCS3) to maintain homeostasis, which is upregulated in the obese condition and contributes to pathology instead, which is paradoxical. Rather than simply suppressing inflammation, the aberrant SOCS3 activity causes the degradation of IRS-1/2 that is basically usurping the negative feedback loop and imposing insulin resistance and a catabolic state [8].

### The protein kinase C family

Protein Kinase C (PKC) isoforms, especially the new isoform PKC-θ serve as lipid-sensitive essential kinases in the pathophysiology of muscle dysfunction. One of the main causes of lipid-induced insulin resistance is the intramyocellular diacylglycerol accumulation that directly activates the PKC-θ. Acting like JNK, activated PKC-θ phosphorylates IRS-1 at inhibitory serine residues, in effect silencing the insulin signal. This pathway thus constitutes a direct molecular connection between the ectopic fat deposition (steatosis) and the skeletal muscle metabolic failure [12].

## The adipose secretome: drivers of metaflammation and atrophy

Adipose tissue in SO is not inert; it is hyperplastic, hypertrophic, and infiltrated by immune cells, resulting in a skewed secretory profile.

### Pro-inflammatory adipokines

The inappropriate release of pro-inflammatory adipokines has an adverse effect on muscle homeostasis in the context of SO. The presence of leptin resistance and hyperleptinemia are widespread; physiological leptin promotes fatty acid oxidation, but its excessive concentration in the state of resistance does not help to reduce lipid overload but on the contrary, contributes to the inflammatory environment. At the same time, Resistin is increased in obesity and has a direct negative effect on glucose uptake in skeletal muscle by preventing phosphorylation of IRS-1 and Akt. It is worth noting that a large ratio of resistin/IGF-1 has been reported as a distinct biomarker to sarcopenia risk in elderly people. Likewise, Visfatin and Chemerin are increased in obesity and are associated with insulin resistance and inflammation, but their particular mechanistic role in muscle atrophy requires further investigation in human systems [12].

### Anti-inflammatory adipokine deficiencies

Deficiencies in protective, anti-inflammatory adipokines are also additional pathogenesis of SO. Adiponectin is an effective insulin-sensitizing hormone that stimulates AMPK and PPAR- α to induce fatty acid oxidation. In SO, however, the levels of adiponectin drop - a condition referred to as hypoadiponectinemia. This vital loss eliminates one of the main inhibitors of muscle inflammation and NF-κB signaling, which exposes the muscle tissue to lipotoxic stress [12]. Likewise, Apelin an adipokine that promotes glucose uptake and mitochondrial biogenesis is present in lower levels in sarcopenic individuals; this deficiency directly leads to the breakdown of the key muscle maintenance and repair pathways [13].

## The skeletal muscle secretome: loss of protective signals

Sarcopenia involves the depletion of the endocrine ability of the muscle. The loss of muscle mass results in a lack of myokines to regulate adipose tissue plasticity.

### Irisin and the browning defect

Irisin, a myokine that is cleaved by the precursor FNDC5 in reaction to muscle contraction, is an important regulator of metabolism, promoting the browning of white adipose tissue by increasing UCP1 [14]. Nonetheless, the resulting atrophy of muscle and sedentary lifestyle of SO cause a significant decrease in the amount of irisin in circulation. This lack greatly affects the non-shivering thermogenesis and energy loss, forming a feedback mechanism that helps to accumulate more fat. In addition, since irisin has autocrine effects that enhance mitochondrial biogenesis, loss of it increases mitochondrial degeneration in the remaining muscle fibers, further complicating the pathology [13].

### Interleukins: the dual role of IL-6 and IL-15

Interleukins have a dual nature in the regulation of metabolic homeostasis especially in the SO context. It has a specific dichotomy in terms of its source and temporal secretion pattern: skeletal muscle-derived IL-6, which is released in transient, high-amplitude pulses during physical contraction, acts as an anti-inflammatory myokine that actively enhances insulin sensitivity [15]. On the other hand, a pro-inflammatory condition is mediated by the chronic, elevated basal IL-6 secreted by obese adipose tissue. While baseline circulating IL-6 is typically low in healthy humans (< 2–3 pg/mL), clinical and murine models demonstrate that subjects with obesity exhibit chronically elevated levels, with an estimated 30% of circulating basal IL-6 originating directly from adipose tissue [16]. In SO, this balance is pathologically disturbed: baseline circulating IL-6 is significantly higher than in obesity or sarcopenia alone, reflecting a compounded inflammatory burden, while the positive myokine IL-6 pulses are severely reduced by physical inactivity [17, 18]. At the same time, IL-15 is an important anabolic myokine that prevents adipogenesis and promotes muscle protein accretion. Nevertheless, its depletion of SO eliminates a natural physiological inhibition of adipose growth, as well as muscle catabolism, which further worsens the disease phenotype [13].

### Novel myokines: FGF-21 and LIF

**Fig. 2:**
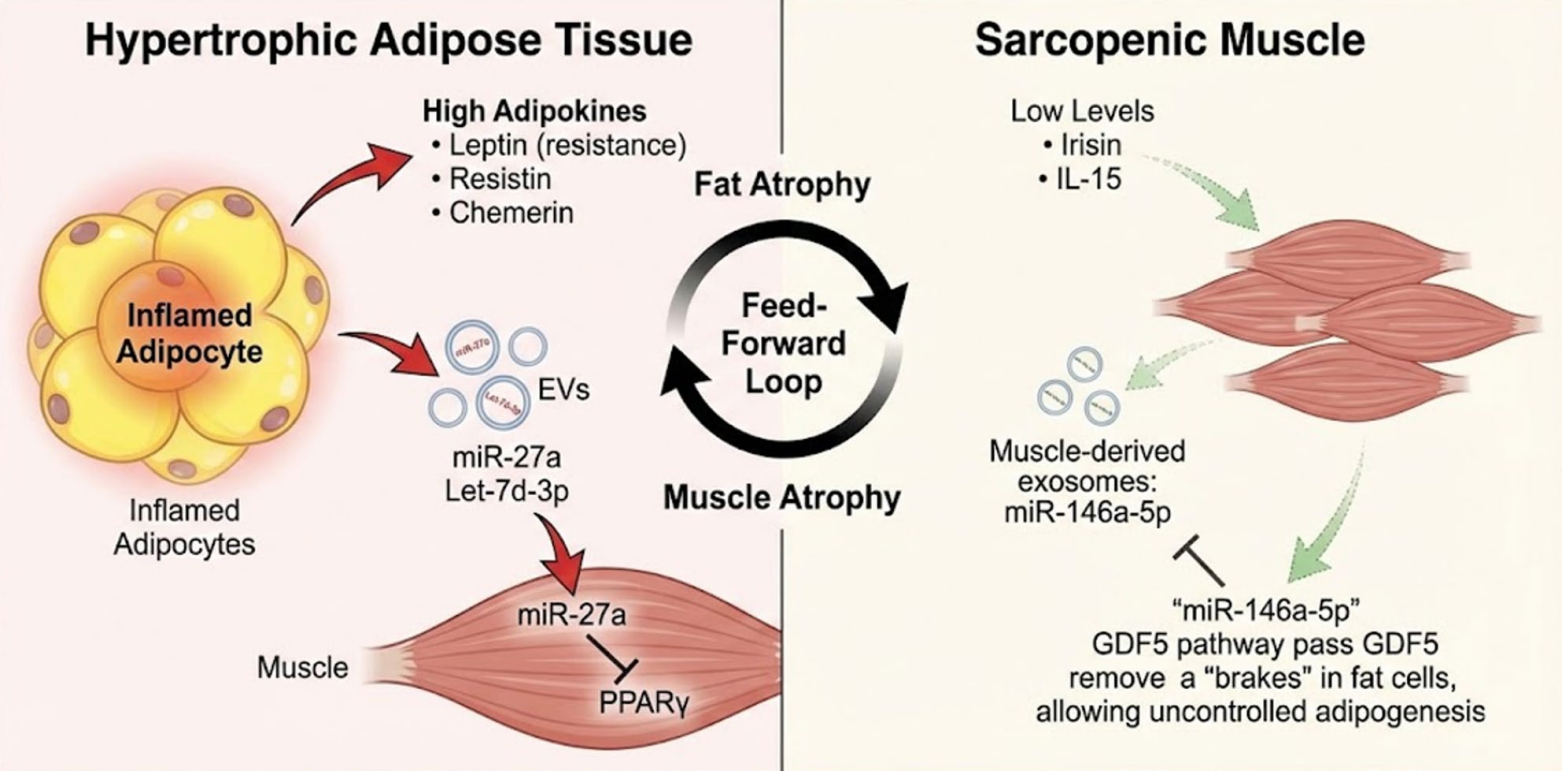
The “Vicious Cycle” of Muscle-Adipose Crosstalk

Fibroblast Growth Factor 21 (FGF-21) plays a crucial role in the regulation of glucose metabolism and energy expenditure, but in SO, the reduction in the levels of FGF-21 is strongly linked to the dysfunction of mitochondria and pathologic whitening of brown adipose tissue. Simultaneously, the suppression of the Leukemia Inhibitory Factor (LIF) cytokine which is fundamental to the proliferation of the satellite cells further impairs the regenerative ability of sarcopenic muscle after injury or mechanical stress [13] Fig. 2.

## Extracellular vesicles and the miRNA regulatory network

The Exosomal miRNA Regulatory Network has been discovered as a paradigm shift in understanding muscle-adipose crosstalk. MicroRNAs are transported by exosomes through intercellular communication between tissues, modifying the gene expression of the recipient cells [19].

### Adipose-to-muscle communication (the atrophic signal)

Obese or aged adipose tissue-derived EVs are essential vectors of specific atrophy-inducing microRNAs (atromiRs) internalized by myocytes to perpetuate pathology. As an example, in murine models of aging, EVs derived from aged perimuscular adipose tissue (PMAT) are loaded with Let-7d-3p, which inhibits the transcription factor HMGA2 in isolated muscle stem cells; the inhibition of this target is harmful, causing stem cell senescence and regenerative failure. Certain miRNAs like miR-27a and miR-155 further worsen the metabolic disruption. Clinical data reveals that miR-27a is highly elevated in the serum of human obese T2DM patients; in vitro studies demonstrate that when miR-27a enters cultured myocytes, it inhibits PPARγ expression, thereby causing insulin resistance and enhancing lipotoxicity. Equally, the miR-155 produced by Adipose Tissue Macrophages targets PPARγ and C/EBP-beta and inhibits the insulin-stimulated glucose uptake and facilitates an inflammatory phenotype [9]. Also, miR-23a is increased in atrophy and regulates TRIM63 (MuRF1) through a feedback loop, which, in the case of SO, is often linked to the inhibition of anabolic targets [20].

### Muscle-to-adipose communication (the metabolic regulator)

Skeletal muscle releases exosomes that actively inhibit adipogenesis. One of the main elements of this regulatory system is miR-146a-5p, a microRNA that is highly expressed in skeletal muscle exosomes, and acts as a negative regulator of adipogenesis by acting on the GDF5- PPARγ signaling axis. In SO the depletion of muscle mass causes a decrease in the pool of miR-146a-5p in circulation, eliminating this inhibitory control, effectively allowing uncontrolled adipocyte hyperplasia and hypertrophy in fact, in vivo genetic knockout of miR-146a-5p specifically in the skeletal muscle of mice spontaneously causes adiposity and metabolic disturbance, which demonstrates its causal role [21]. Moreover, certain myomiRs including miR-133 and miR-206 are essential in the regulation of satellite cell differentiation and muscle regeneration. Their concentrations are significantly changed in SO, and miR-206 becomes one of the possible predictors of the response to strength training interventions [22] Table 1.

**Table 1:** Key miRNAs Governing Muscle-Adipose Crosstalk in Sarcopenic Obesity

miRNA	Origin	Target Tissue	Validated Target	Mechanism in SO Pathology	Reference
**Let-7d-3p**	Aged PMAT EVs	Muscle Stem Cells	*HMGA2*	Impairs satellite cell self-renewal and regeneration.	[9]
**miR-27a**	Obese Adipocytes	Muscle	*PPARγ*	Induces insulin resistance; promotes lipotoxicity.	[9]
**miR-155**	M1 ATM EVs	Muscle	*PPARγ*,* C/EBP-beta*	Blocks glucose uptake; exacerbates inflammation.	[9]
**miR-146a-5p**	Skeletal Muscle	Adipose Tissue	*GDF5*	Loss of this miRNA allows unrestrained adipogenesis.	[21]
**miR-23a**	Adipose/Muscle	Muscle	*TRIM63* (MuRF1)	Modulates proteolysis; dysregulated in atrophy.	[20]
**miR-144-3p**	Circulating	Muscle	*FoxO1*	Novel biomarker associated with low muscle strength.	[38]
**miR-486**	SKM-EVs	Muscle/Adipose	*FoxO1*	Downregulates FoxO1 to inhibit proteolysis (decreased in SO).	[39]

ATM, Adipose Tissue Macrophage; EVs, Extracellular Vesicles; HMGA2, High Mobility Group AT-Hook 2; PMAT, Perimuscular Adipose Tissue; PPARγ, Peroxisome Proliferator-Activated Receptor Gamma; SKM, Skeletal Muscle; SO, Sarcopenic Obesity

## Cellular mechanisms: immune plasticity and organelle dysfunction

The molecular signals described above converge on specific cellular machineries to drive the SO phenotype.

### Macrophage phenotypic switching and metaflammation

**Fig. 3:**
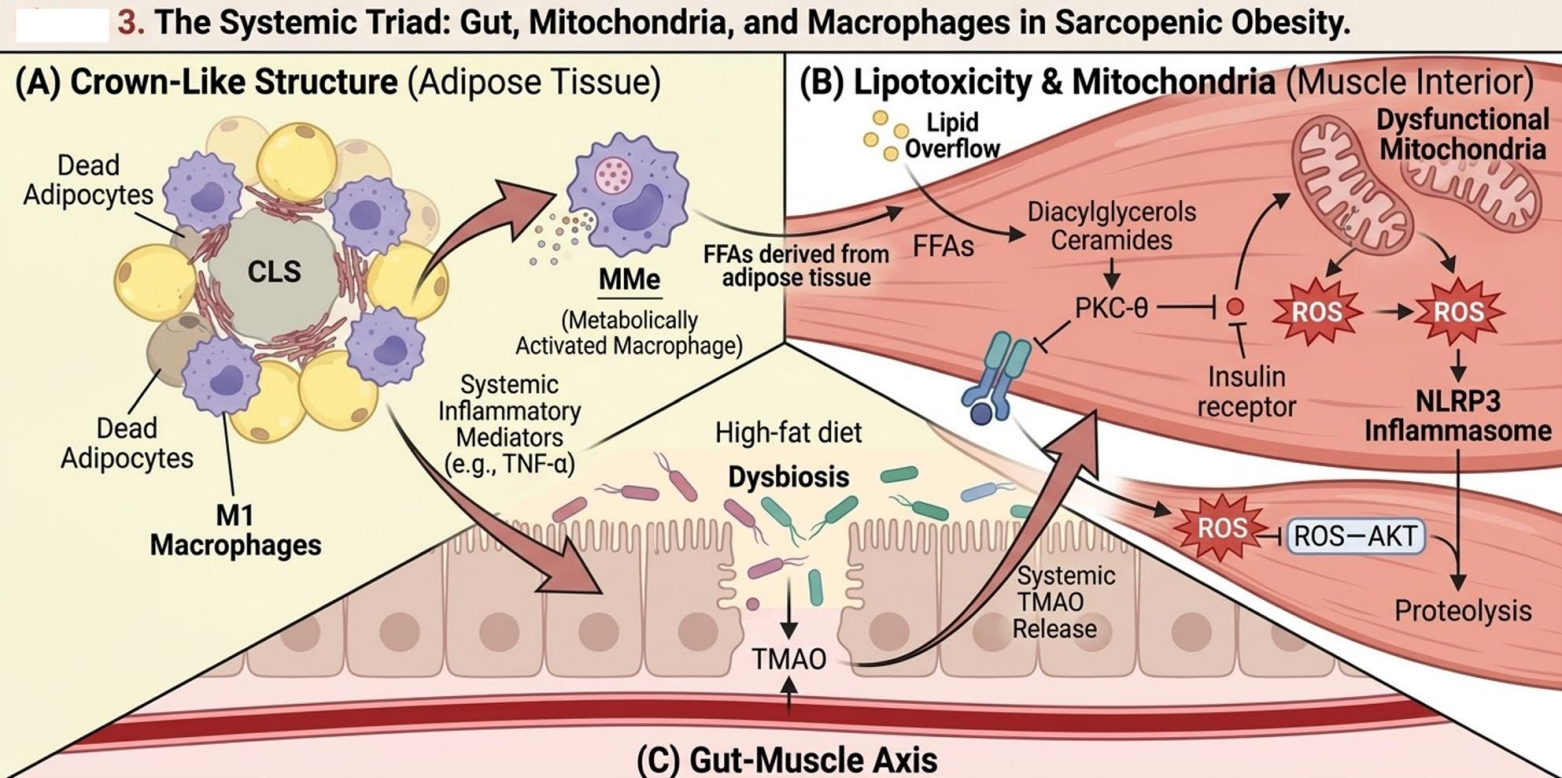
The Systemic Triad: Gut, Mitochondria, and Macrophages in Sarcopenic Obesity. (**A**) shows a Crown-Like Structure in adipose tissue, where MMe surround dead adipocytes, releasing systemic inflammatory mediators (e.g., TNF-α) that directly infiltrate the muscle compartment. (**B**) illustrates Lipid Overflow in muscle, where free fatty acids derived from adipose tissue convert to lipotoxic species that activate PKC-θ, and dysfunctional mitochondria release ROS, triggering the NLRP3 inflammasome. (**C**) depicts the gut-muscle axis, where a high-fat diet causes dysbiosis, leading to systemic TMAO release that circulates to the muscle, activating the ROS-AKT pathway and inducing muscle proteolysis

The immune system is the main system that coordinates metabolic dysfunction, coordinating complicated cellular changes in tissues. In lean adipose tissue, resident Adipose Tissue Macrophages predominantly exhibit an M2-like (anti-inflammatory) phenotype to maintain homeostasis. However, hypoxia and adipocyte stress in obesity induce a pronounced phenotypic transition toward the M1 (pro-inflammatory) state. These M1 macrophages cluster around dead adipocytes to create Crown-Like Structures which release large amounts of TNF-α and IL-1b. Primary investigations have also described a distinct macrophage subset, termed Metabolically Activated Macrophages (MMe), triggered by high glucose and palmitate (CD36+, ABCA1+, PLIN2+) [23]. Unique to obese metabolic condition, MMe cells are pro-inflammatory but execute lysosomal exocytosis to dispose of dead adipocytes, which are major contributors of insulin resistance [24]. There are parallel changes in skeletal muscle macrophages; M1 inflammation leads to acute inflammation, whereas M2-like macrophages in aged muscle can paradoxically cause fibrosis and ectopic fat deposition [25]. Moreover, a skeletal muscle-resident subset (LYVE1^−^/MHCII^low^, FcγRIV^+^/CD36^+^) has also been found, and this might be the one that removes senescent cells, although its activity seems to be overwhelmed in the conditions of SO [26] Fig. 3.

### Mitochondrial dysfunction and lipotoxicity

One of the most important processes that lead to muscle dysfunction is lipotoxicity, in which the leakage of lipids leads to the accumulation of toxic lipid species, including ceramides and diacylglycerols, in muscle cells. These metabolites stimulate PKC-θ and JNK, which have a direct inhibitory effect on insulin signaling [8]. At the same time, sarcopenic muscle has high levels of mitochondrial deterioration, which is lower content and quality as a result of mitophagy failure. This degradation is facilitated by the down-modulation of PGC-1alpha through the AMPK-SIRT1 pathway to cause the accumulation of dysfunctional mitochondria and increased Reactive Oxygen Species (ROS). Thus, ROS trigger NLRP3 inflammasome and stabilize HIF-1alpha, which leads to an inflammatory and proteolytic cycle [27].

### The gut-microbiota-muscle axis

**Fig. 4:**
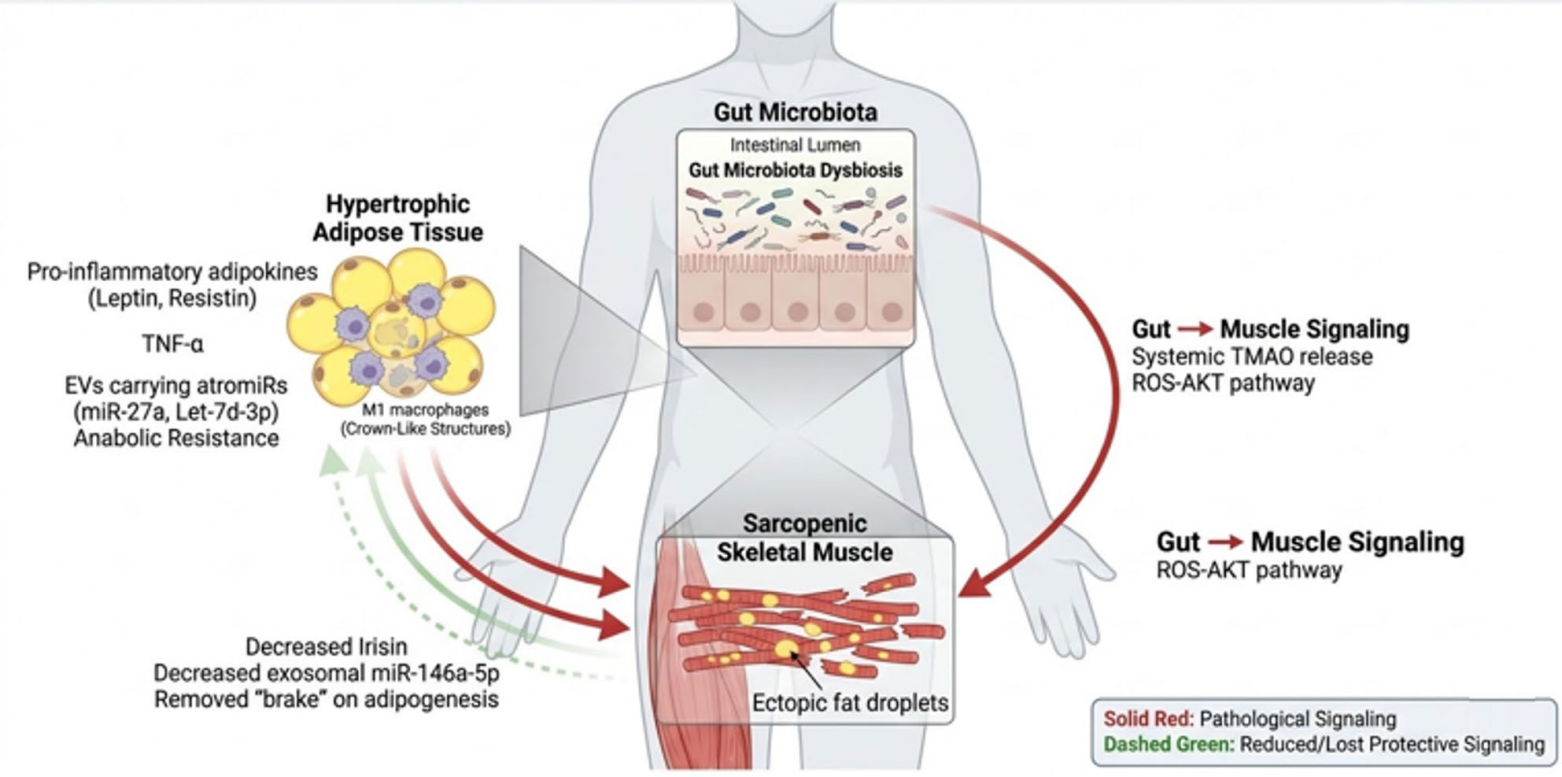
Systemic Crosstalk Network in Sarcopenic Obesity

There is growing evidence that gut dysbiosis is associated with the pathogenesis of SO. Diets rich in fats have been reported to enhance the proliferation of harmful intestinal microbes, which results in the high synthesis of Trimethylamine N-oxide (TMAO). Mechanistically, high levels of serum TMAO exacerbates the SO phenotype by inducing the ROS-AKT/mTOR signaling pathway in skeletal muscle, which also promotes skeletal muscle proteolysis and inhibits skeletal muscle protein synthesis [28]. Collectively, these findings establish the existence of a critical gut-muscle axis that operates in parallel to the established adipose-muscle axis, forming a comprehensive systemic network **(**Fig. 4**)**.

## Experimental models: from genetic mice to organ-on-a-chip

Our understanding of SO relies on a diverse array of experimental models that replicate specific aspects of the human pathology.

### Advanced in vitro systems: organ-on-a-chip

Although classical 2D co-cultures, including C2C12 myoblasts with 3T3-L1 adipocytes have been able to validate that adipocyte secretomes cause atrophy, such models are not always physiologically relevant. To fill this gap, emerging microphysiological systems have been created. As an example, an Intestine-Liver-Muscle Axis Chip has been produced in 3D, pump-free, to simulate the metabolic interaction by using the oscillatory flow of gravity to simulate blood flow between chambers with Caco-2, HepG2, and primary human skeletal myoblasts. This active co-culture had a profound effect on skeletal muscle differentiation-induced by augmented MYH expression- and metabolic activity in contrast to inactive controls, showing that the presence of hemodynamic shear stress and constant metabolite exchange is a requirement to realistic muscle-organ crosstalk modeling [29]. Moreover, 3D bio-fabrication methods are being applied to develop spatially structured muscle-fat interfaces; the models can be used to study direct physical contact and paracrine diffusion gradient, and are capable of recapitulating the intermuscular adipose tissue microenvironment [30].

### Animal models of sarcopenic obesity

The Senescence-Accelerated Mouse Prone 8 (SAMP8) is an important model to study accelerated aging in SO. SAMP8 mice fed a high-fat diet acquire a robust SO phenotype at 20 months, which is severe muscle atrophy, loss of myofibers, and metabolic syndrome. Interestingly, this model is also characterized by the presence of osteosarcopenic obesity that is characterized by the enhancement of chondrocyte apoptosis and cartilage destruction, indicating the central role of muscle-fat crosstalk in bone well-being [31]. In addition to these observations, genetic knockout models are useful to explain the exact molecular processes underlying SO. A recent study showed the critical role of cap-dependent translation in preserving muscle mass during metabolic stress is the double knockout of translational repressors 4E-BP1/2 (4EBP1/2 DKO) [32]. In the same way, the lack of superoxide dismutase causes an increase in oxidative stress and muscle atrophy, which is effectively similar to the mitochondrial impairment of SO. Also, the miR-146a knockout in adipose tissue directly demonstrates the presence of crosstalk through EVs, because the loss of this microRNA in adipose tissue alone leads to muscle wasting in the whole body and decreased exercise capacity [33].

## Clinical implications and therapeutic frontiers

### The GLP-1 receptor agonist paradox

With the emergence of GLP-1 receptor agonists such as semaglutide and tirzepatide, the management of obesity has changed but has brought about a new clinical dilemma. Although they are useful in fat loss, clinical trials, including STEP 1 [34] and SURMOUNT-1 [35], suggest that 20–50% of the weight lost is lean body mass, which is a cause of concern in relation to iatrogenic sarcopenia. The above observation has a mechanistic paradox, because preclinical evidence indicates that GLP-1 signaling is protective; activation of GLP-1 receptors or indirect metabolic ameliorations activates the AMPK-SIRT1-PGC1 alpha axis and suppresses NF- kB, which in principle should prevent atrophy [36]. In turn, the muscle atrophy that is being seen in humans is probably due to the speed of weight loss and caloric deprivation, namely, gravitational unloading, and not to direct catabolic toxicity, a disconnection that requires the creation of combination therapies to maintain muscle mass.

### Emerging therapeutics

A number of new therapeutic approaches are under testing to overcome the problem of muscle wastage during a rapid weight loss. In the lead are combination therapies, in which the co-administration of GLP-1 receptor agonists with myostatin inhibitors (e.g., bimagrumab) or soluble ActRIIB decoys are under investigation to successfully uncouple weight loss and muscle loss [37]. At the same time, miRNA therapeutics provide a precision medicine by attacking the Exosomal miRNA Regulatory Network; such as the introduction of miR-146a-5p mimics using exosomes has been demonstrated to reverse the effects of high-fat diet-induced obesity and muscle wasting in mice [21], while anti-miR-27a strategies could potentially ameliorate insulin resistance [9]. Also, exercise mimetics - compounds like AICAR that are able to trigger the irisin pathway or AMPK - are under development to mimic the positive secretome of exercise, and so induce browning and protein synthesis in even specific populations incapable of exercise [12].

## Conclusion

SO is a complex breakdown of systemic communication. It is motivated by a vicious cycle in which inflamed adipose tissue releases atrophic factors (TNF-α, myostatin, miR-27a) through cytokines and extracellular vesicles, which directly steal the transcriptional machinery of the muscle to prevent regeneration and induce proteolysis. At the same time, the atrophied muscle does not give the metabolic signals (irisin, miR-146a) required to suppress adiposity.

This comprehensive overview points out that the future of SO management is in reinstating this molecular dialogue. The most promising way to go is the integration of novel biomarkers (exosomal miRNAs), the development of advanced 3D human models to screen drugs and the application of targeted therapies that address the specific molecular deficits of the sarcopenic obese patient, as opposed to treating obesity and sarcopenia as distinct entities. The evidence highlights that we should not be concerned only with the mass but with the quality of the molecules and communicative ability of the musculoskeletal system.

## Data Availability

Not applicable.

## Abbreviations

4E-BP1: Eukaryotic translation initiation factor 4e-binding protein 1
ActRIIB: Activin receptor type IIB
AMPK: AMP-activated protein kinase
ATMs: Adipose tissue macrophages
CLS: Crown-like structures
EVs: Extracellular vesicles
FGF-21: Fibroblast growth factor 21
FoxO: Forkhead box O
GLP-1 RAs: Glucagon-like peptide-1 receptor agonists
HMGA2: High mobility group AT-hook 2
IGF-1: Insulin-like growth factor 1
IKK: IκB kinase
IL-6: Interleukin-6
IRS-1: Insulin receptor substrate-1
JAK: Janus kinase
LIF: Leukemia inhibitory factor
MAFbx: Muscle atrophy F-box (also known as Atrogin-1)
miRNA: MicroRNA
MMe: Metabolically activated macrophage
MPS: Microphysiological systems
mTOR: Mechanistic target of rapamycin
mTORC1: Mechanistic target of rapamycin complex 1
MuRF1: Muscle RING-finger protein-1
NF-κB: Nuclear factor kappa B
p70S6K: p70S6 Kinase
PI3K: Phosphoinositide 3-kinase
PKC: Protein Kinase C
PMAT: Perimuscular adipose tissue
ROS: Reactive oxygen species
SAMP8: Senescence-accelerated mouse prone 8
SKM-Exos: Skeletal muscle exosomes
SO: Sarcopenic obesity
SOCS: Suppressors of cytokine signaling
STAT: Signal transducer and activator of transcription
TMAO: Trimethylamine N-oxide
TNF-α: Tumor necrosis factor-alpha
